# Induction of Necrosis in Human Macrophage Cell Lines by *Corynebacterium diphtheriae* and *Corynebacterium ulcerans* Strains Isolated from Fatal Cases of Systemic Infections

**DOI:** 10.3390/ijms20174109

**Published:** 2019-08-22

**Authors:** Dulanthi Weerasekera, Jonas Hahn, Martin Herrmann, Andreas Burkovski

**Affiliations:** 1 Department Biologie, Friedrich-Alexander-Universität Erlangen-Nürnberg, 91058 Erlangen, Germany; 2 Universitätsklinikum Erlangen, Friedrich-Alexander-Universität Erlangen-Nürnberg, 91054 Erlangen, Germany

**Keywords:** cell death, FACS, host-pathogen interaction, live cell imaging, macrophages, THP-1 cells

## Abstract

When infecting a human host, *Corynebacterium diphtheriae* and *Corynebacterium ulcerans* are able to impair macrophage maturation and induce cell death. However, the underlying molecular mechanisms are not well understood. As a framework for this project, a combination of fluorescence microscopy, cytotoxicity assays, live cell imaging, and fluorescence-activated cell sorting was applied to understand the pathogenicity of two *Corynebacterium* strains isolated from fatal cases of systemic infections. The results showed a clear cytotoxic effect of the bacteria. The observed survival of the pathogens in macrophages and, subsequent, necrotic lysis of cells may be mechanisms explaining dissemination of *C. diphtheriae* and *C. ulcerans* to distant organs in the body.

## 1. Introduction

From the 90 *Corynebacterium* species described in 2014, approximately 50 species were initially isolated from humans or human clinical material [1]. Many of these species rarely cause infections, however, a few potent human pathogens are found within this group. Among these species, the most prominent is *Corynebacterium diphtheriae*, the causative agent of diphtheria.

Diphtheria is a paradigm of an infectious disease caused by toxigenic bacteria, which when untreated often results in a fatal outcome due to the detrimental effects of diphtheria toxin (for review, see [2,3]). Classical diphtheria of the upper respiratory tract is transmitted from human to human by respiratory droplets. It is characterized by a putrid smell and by a thick, dirty-grayish or brownish pseudomembrane, which renders breathing difficult. At the beginning of the 20th century the introduction of mass vaccination with diphtheria toxoid vaccine led to a sharp decline of diphtheria cases (for review, see [4]). However, the disease was never fully eradicated and to date a few thousand cases are still reported to the World Health Organization every year [5,6]. Due to this persistence and an overall case-fatality rate of 5% to 10%, with higher death rates of up to 20% among patients younger than 5 years and in unprotected patients, *C. diphtheriae* is still present on the list of the most important global death-provoking pathogens [7]. Nowadays, this ignominious role is challenged by a taxonomically closely related emerging pathogen, *Corynebacterium ulcerans*, which was first described by Gilbert and Stewart, who isolated this bacterium from the throat of a patient with diphtheria-like symptoms [8]. In fact, when lysogenized by a *tox* gene-carrying corynephage, *C. ulcerans* can also produce diphtheria toxin. During the last years, human infections associated with *C. ulcerans*, which can often be ascribed to zoonotic transmission, appear to be rising in European countries and have in fact outnumbered diphtheria cases caused by toxigenic *C. diphtheriae* [9,10,11,12].

Interestingly, besides classical respiratory or cutaneous diphtheria, various cases of systemic infections were described for the two species including arthritis, endocarditis, osteomyelitis, pneumonia, and others [13,14,15]. These observations may indicate that the two species cannot only successfully colonize epithelia, but also spread within the body and infiltrate tissues that are a considerable distance from the primary infection site.

In this study, two corynebacterial strains isolated from fatal cases of infection were studied, *C. diphtheriae* HC04 and *C. ulcerans* 809. *C. diphtheriae* HC04 was isolated from the catheter of a 7 years old girl. The patient developed complications including arthritis, myositis, peripheral, and central nervous system (CNS) emboli, as well as microaneurysm with brain hemorrhage and died due to septic shock caused by endocarditis [13,14,15]. *C. ulcerans* 809 was isolated from a sample of bronchoalveolar lavage (BAL) from an 80 year old woman, diagnosed with pulmonary infections and chronic limb ulcers. Post-mortem medical examinations revealed evidence of multiple organ failures and it was concluded that the unusual nature of the pathogen possibly contributed to the death of the patient [16,17]. While the two strains are non-toxigenic with respect to the diphtheria toxin, they unfortunately successfully interacted with the human innate immune system. A critical point in this respect is the interaction of phagocytic cells with invading bacteria, which typically ends with the elimination of the microorganisms from the human body.

Escape mechanisms of pathogenic corynebacteria with respect to host defense systems are generally not well understood. On the basis of preliminary experiments, induction of apoptosis and necrosis in human macrophages was suggested for *C. diphtheriae* strains [18], while induction of apoptosis was excluded for *C. ulcerans* [19]. However, the outcome of such experiments was critically dependent on the duration of infection and the number of pathogenic bacteria challenging the immune system. Therefore, a detailed characterization of macrophage interaction with these two strains was carried out utilizing a combination of live cell imaging and fluorescence-activated cell sorting (FACS) analyses.

## 2. Results

### 2.1. Species-Specific Proliferation and Elimination of Corynebacteria Inside Human Macrophages

As a critical parameter for the outcome of pathogen-host interactions, the behavior of the two strains with respect to endocytosis and survival in THP-1 cells was tested. Previous studies indicated a deleterious effect at multiplicities of infection (MOI) higher than 50 for *C. ulcerans* [12] and *C. diphtheriae* strains [20,21] on human macrophage-like cells.

**Figure 1:**
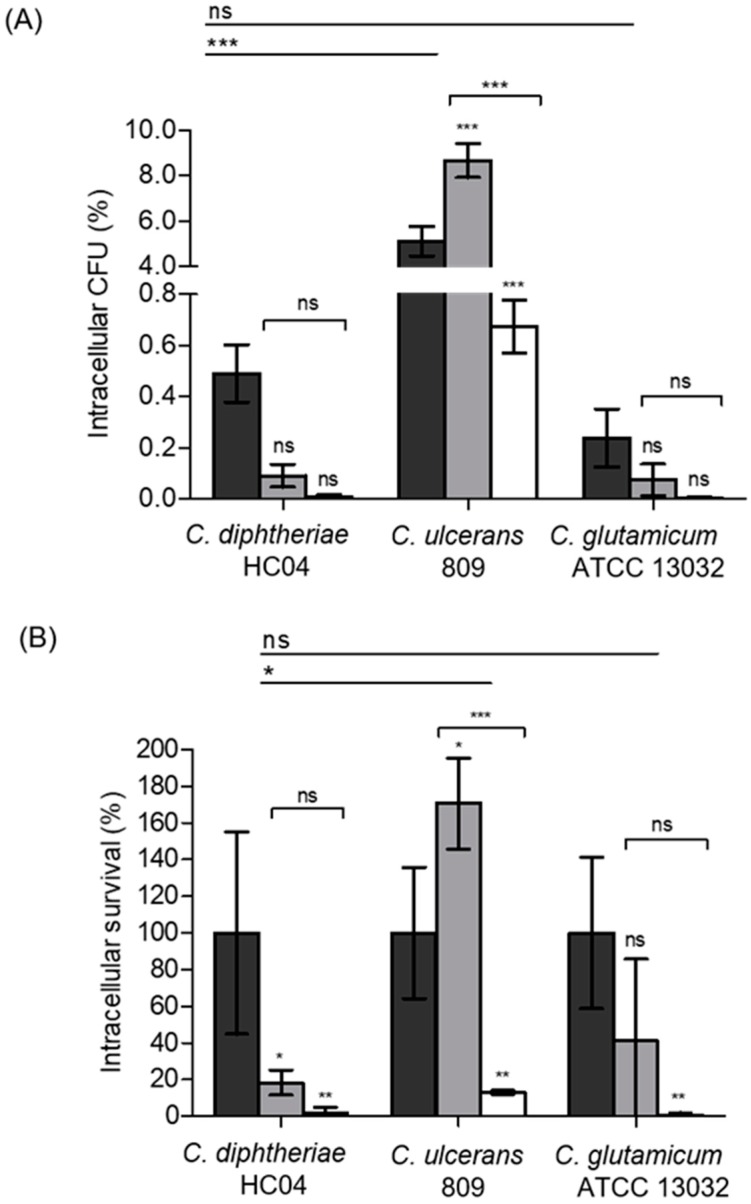
Quantitative analysis of viable intracellular corynebacteria in THP-1 cells. THP-1 cells were infected with *C. diphtheriae* HC04, *C. ulcerans* 809, and *C. glutamicum* ATCC 13032 at a multiplicities of infection (MOI) of 25 for 30 min. Extracellular bacteria were killed by gentamicin treatment and intracellular colony-forming units (CFUs) were recovered on blood agar plates by plating the lysates of the harvested cells. The percentages of viable intracellular bacteria at 2 h (black bars), 8 h (grey bars), and 20 h (white bars) are shown. (**A**) The percentage of intracellular CFU with respect to the applied inoculum and (**B**) the percentage of intracellular survival of bacteria relative to the value of 2 h post-infection set to 100%. Data shown are mean ± standard deviation values of at least three independent biological replicates each performed in triplicates. Statistically relevant differences based on ANOVA values below 0.05, 0.01, and 0.001 are indicated by one, two, and three asterisks, respectively.

When a MOI of 25 was used 2 h post-infection, approximately 0.5% of the initially applied colony-forming units were detected for *C. diphtheriae* HC04, whereas, a significant higher rate of about 5% was observed for *C. ulcerans* 809. The non-pathogenic *C. glutamicum* ATCC 13032 used as a control showed the lowest number of colony-forming units with approximately 0.2%. The number of bacteria appeared to be constantly declining within 20 h for *C. diphtheriae* HC04 and *C. glutamicum* ATCC 13032. In contrast, an increase of the number of intracellular *C. ulcerans* within macrophages was observed at 8 h post-infection, while after 20 h a significant reduction to 0.7% was found (Figure 1A).

When the number of colony-forming units (CFUs) at 2 h post-infection was set to 100%, a steady decline of the survival rate of *C. diphtheriae* and *C. glutamicum* was detected, while significant growth was observed for *C. ulcerans* at 8 h post-infection, when the bacteria reached 171 ± 48% survival rate. However, also in this case, the number of bacteria 20 h post-infection was drastically reduced reaching a survival rate of 13 ± 2% (Figure 1B).

### 2.2. Infection with C. diphtheriae and C. ulcerans Results in Condensation of DNA in Macrophage Nuclei and Induces Cell Lysis

**Figure 2:**
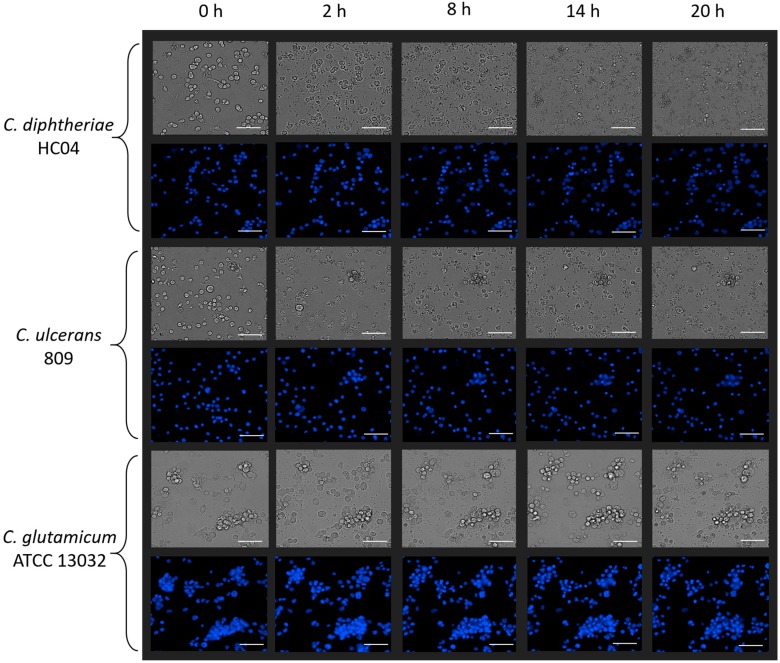
Smaller and brighter nuclei and partial detachment of cells reflect corynebacteria induced cell damage. Human macrophage cells (THP-1) were infected with pathogenic *C. diphtheriae* HC04, *C. ulcerans* 809, and non-pathogenic *C. glutamicum* ATCC 13032 at a MOI of 25. The nuclei of THP-1 cells were monitored at different stages of infection by live-imaging microscopy with Hoechst 33342 nuclear stain and analyzed with ImageJ software. Scale bars indicate 100 µm.

The observed uptake and elimination of the *Corynebacterium* species tested indicated a fully functional defense of the cells against the bacteria. However, microscopic inspection of the cell cultures gave a more differentiated picture. In contrast to *C. glutamicum* ATCC13032, contact with *C. diphtheriae* HC04 and *C. ulcerans* 809 led to detrimental effects on cell cultures. Depending on the time of incubation, progressive detachment of macrophages was observed as described before [12,21] (Figure 2).

**Figure 3:**
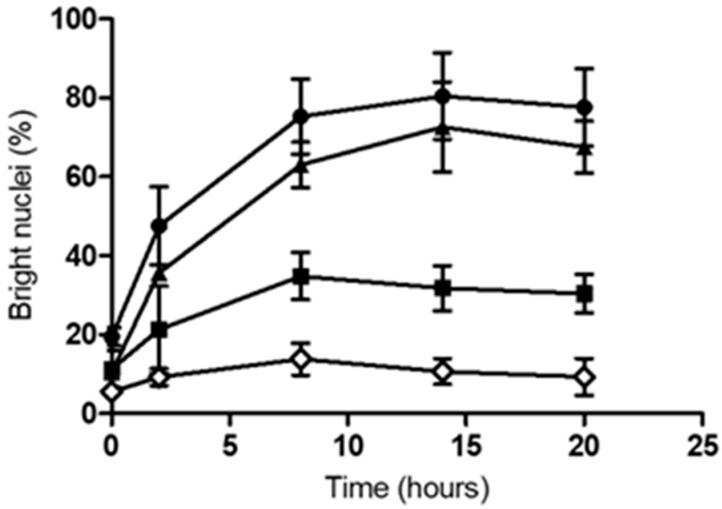
Quantitative analysis of smaller and brighter nuclei at different time points upon infection with corynebacteria. Human THP-1 macrophage cells were infected with pathogenic *C. ulcerans* 809 (●), *C. diphtheriae* HC04 (▲), and *C. glutamicum* ATCC 13032 (■) at a MOI of 25. Uninfected cells (◊) served as the control group. Data shown are the mean values of at least three independent biological replicates each performed in triplicate ± standard deviations.

Fluorescence microscopy using Hoechst nuclear stain [22] also revealed bacteria-induced damage to the human macrophages. Upon contact with pathogenic corynebacteria, DNA was successively condensing over time and nuclei appeared smaller and brighter as compared with cells, which had contact with non-pathogenic *C. glutamicum* (Figure 2). While the number of bright nuclei increased gradually during the course of infection by *C. diphtheriae* HC04 to about 70% and *C. ulcerans* 809 to 80%, only 30% increase was shown at 8 h post-infection by the non-pathogenic *C. glutamicum* ATCC 13032 that remained constant at later time points. The bright nuclei of the uninfected cells only slightly increased to about 13% (Figure 3).

**Figure 4:**
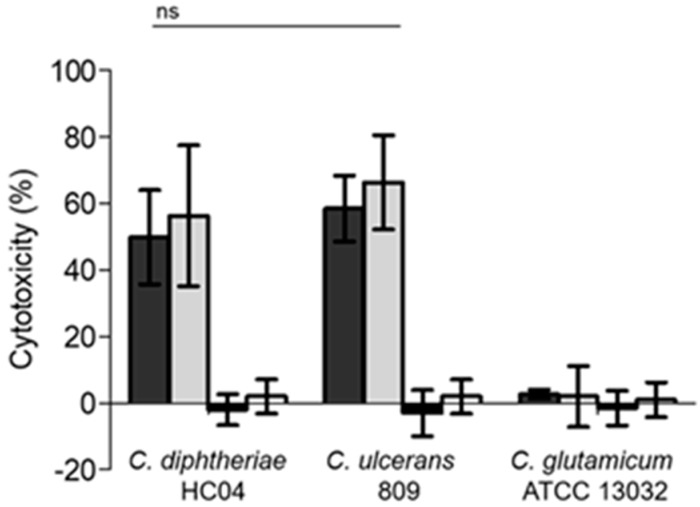
Lactate dehydrogenase release by THP1-blue cells upon infection with corynebacteria. The release of lactate dehydrogenase from THP1-blue cells was measured as marker of host cell damage due to infection with pathogenic corynebacteria. Cells were infected with *C. diphtheriae* HC04, *C. ulcerans* 809, and *C. glutamicum* ATCC 13032 for 20 h. Grey bars indicate infection with live bacteria, black and white bars show results of UV-killed bacteria. Dark grey and black bars indicate a MOI of 1 and light grey and white bars indicate a MOI of 10. Data shown are mean values of at least three independent biological replicates each performed in triplicates ± standard deviation.

As an independent quantitative assay to verify the observed detrimental effects, release of lactate dehydrogenase (LDH) from the cells in response to contact with corynebacteria was measured. While cultures of non-pathogenic *C. glutamicum* ATCC 13032 led to no significant LDH release into the supernatant, cell cultures incubated with *C. diphtheriae* HC04 and *C. ulcerans* 809 showed high LDH activity dependent on the MOI applied. Interestingly, no effect was observed when UV-inactivated corynebacteria were applied indicating an active mechanism of *C. diphtheriae* and *C. ulcerans* induced damage (Figure 4).

### 2.3. Detrimental Effects Depend on Endocytosis of C. diphtheriae HC04 and C. ulcerans 809 and Bacterial Replication within Macrophages

**Figure 5:**
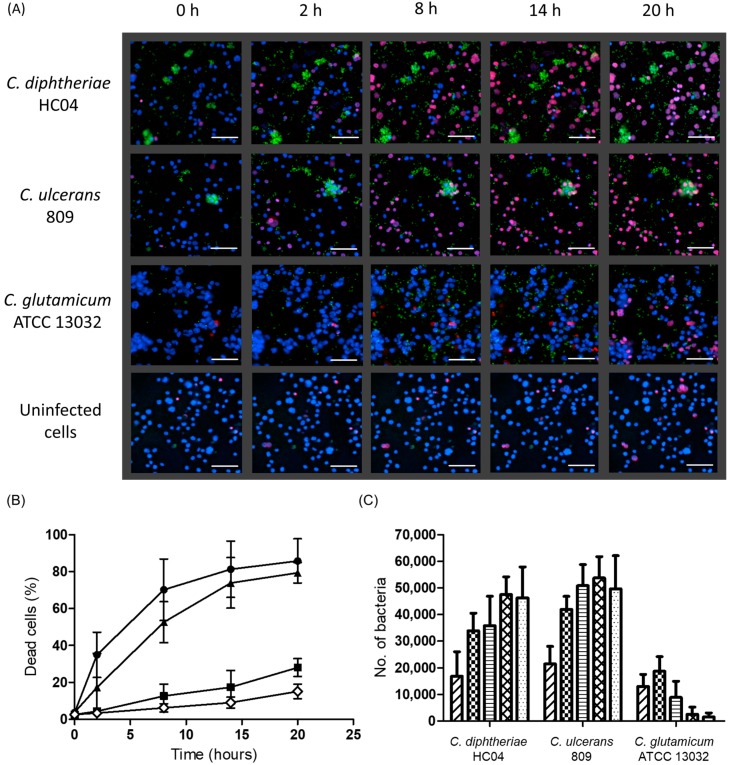
Fluorescence live cell imaging of THP-1 cells infected with green-fluorescent protein (GFP) labelled bacteria. (**A**) THP-1 cells were stained with Hoechst and propidium iodide (PI) 20 min prior to infection with *C. diphtheriae* HC04 pEPR1p45gfp, *C. ulcerans* 809 pEPR1p45gfp, and *C. glutamicum* ATCC 13032 pEPR1p45gfp at a MOI of 25. The images were captured at different time points and analyzed with Photoshop CS5 and ImageJ software. Scale bars indicate 100 µm. Experiments were carried out independently in triplicate and typical results are shown. (**B**) Quantitative analysis of number of dead cells. The number of dead cells upon infection with *C. diphtheriae* HC04 (▲), *C. ulcerans* 809 (●), and *C. glutamicum* ATCC 13032 (■) are shown. Uninfected cells (◊) served as the control group. (**C**) Quantitative analysis of bacterial replication and growth. The bacterial replication at 0 h (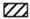), 2 h (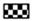), 8 h (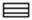), 14 h (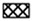), and 20 h (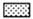) are shown. Data shown are the mean values of at least three independent biological replicates each performed in triplicate ± standard deviations.

For a better resolution of time-dependent effects of corynebacteria-induced cell damage, live cell imaging experiments were carried out for 20 h. A fast increase of the rate of dead cells was observed in the case of *C. diphtheriae* and *C. ulcerans* infection. At 14 h, almost all cells were already dead. In contrast, only a marginal increase in the number of dead cells was observed in the case of *C. glutamicum* infected and uninfected cells reaching approximately 30% and 15%, respectively, after 20 h (Figure 5A,B). In parallel, there is an increase in the number of bacteria in the case of *C. diphtheriae* and, especially, in the case of *C. ulcerans*, while in the case of *C. glutamicum* only a minor increase was observed 2 h after infection (Figure 5C). Interestingly, as in the case of the replication assay (Figure 1), the number of *C. ulcerans* in close proximity to or inside the macrophages was higher as compared to *C. diphtheriae*. In this case, bacterial clusters were often unconnected to the cells.

### 2.4. Induction of Necrosis in Human Macrophages by C. diphtheriae and C. ulcerans

**Figure 6:**
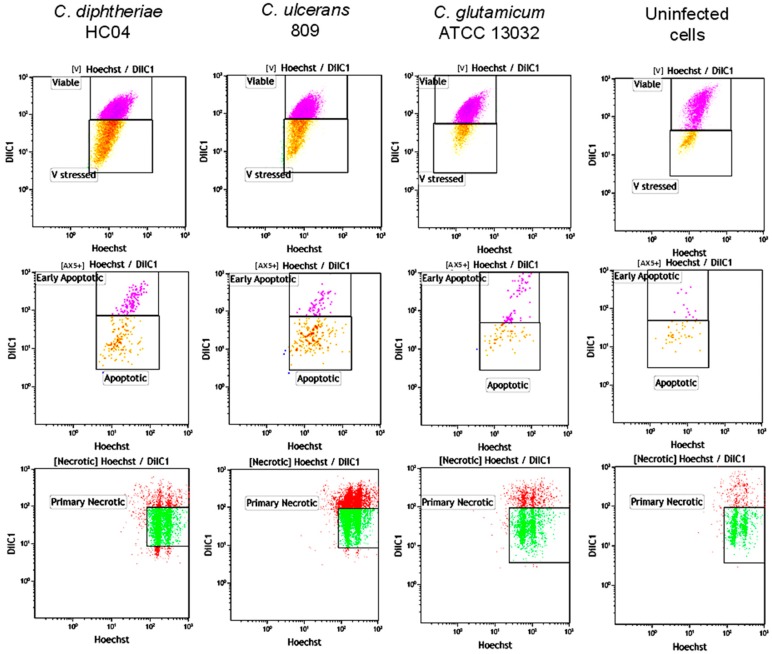
Fluorescence-activated cell sorting (FACS) analysis of THP-1 cells infected with bacteria. THP-1 cells were infected with pathogenic *C. diphtheriae* HC04, *C. ulcerans* 809 and non-pathogenic *C. glutamicum* ATCC 13032 at a MOI of 25. Uninfected cells served as a negative control. After 5 h, cells were harvested and stained with PI to detect dead and damaged cells, Hoechst 33342 to detect the nucleus of healthy living cells, DilC for active mitochondrial membrane potentials, and Annexin V-FITC to detect apoptosis. Experiments were carried out independently in triplicate and typical results are shown.

Death of cells may be induced by different mechanisms with apoptosis and necrosis being the most prominent. In fact, the two mechanisms were previously discussed in [18,19]. Necrosis is a degenerative phenomenon that follows irreversible injury where the cells may lose their membrane integrity at almost all stages of dying [23,24]. Primary necrosis is characterized by rapid plasma membrane rupture, which has often been described as a consequence of extreme stress and thus is considered accidental [23]. Necroptosis is defined as the programmed form of necrosis where the rupture of the plasma membrane results in expulsion of cellular contents into the extracellular space as in all forms of necrotic cell death processes. However, unlike necrosis, during necroptosis, the permeabilization of the cell membrane is tightly regulated. In this study, a combination of different stains was utilized to determine the different stages of cell death and associated changes such as viable, stressed, early or late apoptosis, and primary or secondary necrosis due to corynebacterial infection. While the plasma membrane damage causing phosphatidyl serine exposure was determined with Annexin V-FITC (AxA5-FITC) and propidium iodide (PI) stains, the mitochondrial membrane potential and nuclear DNA content were determined with DilC and Hoechst 33342 nuclear stains (Figure 6). Furthermore, to verify the corynebacteria-induced cell death, the attenuation of the necroptosis was determined by treating the cells with the inhibitor of receptor-interacting protein kinase 1 (RIPK1) prior to infection.

**Figure 7:**
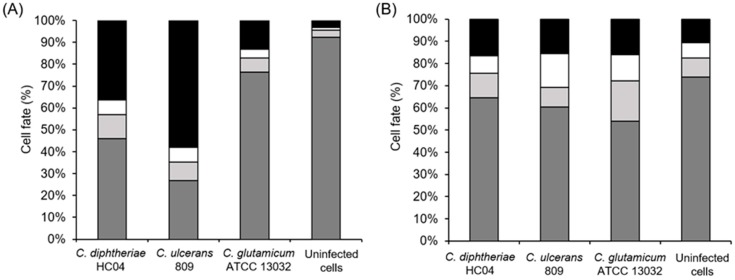
Induction of necrosis. (**A**) The quantified analysis of the fate of THP-1 cells after infection with bacteria. Viable (dark grey bars), stressed (light grey bars), apoptotic (white bars), and necrotic (black bars) cells were quantified using Kaluza Software version 2.1 (Beckman Coulter, Fullerton, CA, USA). (**B**) The fate of the cells with the addition of 50 µM of necrostatin-1 (NEC-1) inhibitor prior to infection with bacteria. The quantified analysis of viable (dark grey bars), stressed (light grey bars), apoptotic (white bars), and necrotic (black bars) cells are shown. Data shown are mean values of at least three independent biological replicates each performed in triplicate.

Quantitative analyses of the FACS data revealed that after 5 h of infection with a MOI of 25 in the case of *C. diphtheriae* HC04, approximately 37% of cells were necrotic, 5% were apoptotic, 46% were viable, and 12% were stressed. In the case of *C. ulcerans* 809, the damage was more pronounced with 59% necrotic, 5% apoptotic, 10% stressed, and 26% viable cells. Contact with *C. glutamicum* ATCC 13032 led to minor cell damage with approximately 14% necrotic, 3% apoptotic, 5% stressed, and 78% fully viable cells, while uninfected macrophages showed more than 90% viable cells with minor rates of necrotic, apoptotic, and stressed cells (3%, 1%, and 3%, respectively) (Figure 7A). When treated with necroptosis inhibitor NEC1, corynebacteria-induced damage was rather similar for pathogenic and non-pathogenic bacteria. Necrosis reached approximately 10% in all cases, 7% apoptotic cells were observed upon contact with *C. diphtheriae*, 18% for *C. ulcerans* infected cells, 12% for *C. glutamicum* infected cells, and 5% for uninfected cells (Figure 7B). Taken together, these experiments favor necrosis as the main cell death mechanism induced by pathogenic corynebacteria under the experimental conditions applied.

## 3. Discussion

The infection of human macrophage-like THP-1 cells with *C. diphtheriae* HC04 and *C. ulcerans* 809 is characterized by two possible outcomes, either the bacteria are eliminated by the phagocytes or the macrophages are damaged by the bacteria. Survival of the pathogens in the macrophages and, subsequent, necrotic lysis of cells may be mechanisms explaining the dissemination of *C. diphtheriae* and *C. ulcerans* to distant organs in the body in the case of systemic infections. In this case, after being internalized by macrophages, bacteria can block phagosome lysosome maturation (this study and [25]), thereby avoiding killing and allowing sustained survival within these cells. Since phagocytes have the ability to migrate through tissues and can be distributed within the body by the blood stream, bacteria may be disseminated inside their host and later released in parts of the body distant to the infection site after killing the macrophages.

The mechanism of induction of necrosis by *C. diphtheriae* and *C. ulcerans* remains unclear, since it is not strictly correlated to the presence of various key virulence factors of these pathogens. Action of diphtheria toxin, the most prominent virulence factor of corynebacteria, can be excluded for the strains studied here, since these are not carrying the *tox* gene [17,26]. This result is consistent with previous interaction studies [18] and a recent publication, which showed no major difference in interaction with macrophages by toxigenic and non-toxigenic *C. diphtheriae* strains [25]. The ribosome-binding protein with similarity to Shiga-like toxins described for strain 809 [17] also can be excluded as the sole inducer of necrosis, since Hacker et al. also showed that the strain, BR-AD22, which is lacking in this toxin, may induce cell death in macrophages [19]. Phospholipase D (PLD) is the major virulence determinant of *Corynebacterium pseudotuberculosis* and is also found in *C. ulcerans* 809 [12,17,19,27]. In *C. pseudotuberculosis*, PLD is involved in the dissemination of the bacteria from the initial infection sites to secondary sites within the host [28,29]. Furthermore, PLD seems to be involved in killing macrophages, since a corresponding mutant strain caused significantly less cell death in murine J774 macrophages as compared to the wild type [30]. Several mechanisms were proposed for the reduction of macrophage viability by PLD. First, this effect may be mediated by a reduction of the integrity of the macrophage plasma membrane as a result of the sphingomyelinase activity of PLD. Since sphingomyelin is located in the outer layer of the plasma membrane, its effect may be mediated by an extracellular rather than intracellular mechanism. Following the death of a macrophage, its cellular content is released and, then, PLD may attack sphingomyelin located in the outer membrane of still viable macrophages. Sphingomyelin is also a major phospholipid component of murine phagosomal membranes. Therefore, the effect of PLD on macrophage viability may also be mediated by reducing the integrity of intracellular compartments, potentially allowing the escape of bacteria from the phagolysosome. As a third mechanism, the actions of PLD within the macrophages may be primarily mediated through disruption of mammalian signaling pathways. Mammalian cells possess two PLD proteins that are involved in cell signaling, in addition to membrane remodeling [31]. However, this characteristic virulence factor of *C. ulcerans* is absent in all *C. diphtheriae* strains known so far, including HC04. In pathogenic mycobacteria, glycolipids such as trehalosyldimycolates play a key role in macrophage interaction [32] and also corynebacterial mycolic acid preparations induce an immune response in primary mouse bone marrow-derived macrophages [33]. However, with respect to the detrimental effects on human macrophage cell lines, no difference was observed between wild-type strains and mycolic acids lacking the *C. diphtheriae* strain [25].

Thus far, it can be concluded that induction of necrosis is an active process as LDH release was only observed in the presence of living bacteria, while dead bacteria had no detrimental effect. Since cytotoxic effects can also be observed with sterile-filtrated culture supernatants [21], secretion of an effector molecule may be a possible mechanism. The identification of such effectors may be a demanding task, since interaction of pathogenic corynebacteria with host cells seems to be very often a multifactorial process [15,19,34,35]. In addition, detection of putative toxins may be difficult due to a low abundance of the proteins as shown for Shiga-like toxins in *C. diphtheriae* and *C. ulcerans* [21,27].

Although *C. ulcerans* 809 was proliferating inside macrophages and, as *C. diphtheriae* HC04, were able to induce necrosis in macrophages, the bacteria were almost completely eliminated at the end of the experiments. This process is initiated by the induction of the inflammatory response. As shown recently, *C. diphtheriae* [20] and *C. ulcerans* [19] induce the NFκB pathway and excretion of proinflammatory cytokines in human THP-1 macrophages. Only when present at a MOI of 50 and higher, *C. diphtheriae* and *C. ulcerans* can overcome this response and kill the majority of macrophages [19,21].

In summary, *C. diphtheriae* and *C. ulcerans* seem to use a similar strategy in host interaction, although at least the tested *C. ulcerans* strains seem to be more readily taken up by macrophages and were more aggressive towards human cells as compared to *C. diphtheriae*. A possible explanation might be a different evolution of host-pathogen interaction. *C. diphtheriae* is almost completely restricted to human hosts, while *C. ulcerans* is widely distributed among different animals [36] and infections of humans are rare.

## 4. Materials and Methods

### 4.1. Bacterial Strains and Culture Conditions

The bacterial strains and plasmids used in this study are listed in Table 1. Heart Infusion (HI) was used to grow all corynebacterial strains used in this study. When appropriate, 50 μg ml^−1^ kanamycin was added to the medium. Electrocompetent corynebacterial strains were transformed with pEPR1-p45gfp plasmid to generate green-fluorescent protein (GFP) expressing bacteria and the positive clones grown on HI agar containing kanamycin were selected. These GFP-expressing bacteria were used for the fluorescence microscopy studies.

### 4.2. Phagocytosis and Survival Assay

Human THP1 monocyte-derived macrophages were cultured in Roswell Park Memorial Institute (RPMI) 1640 medium supplemented with 10% fetal calf serum (FCS), 100 U mL^−1^ penicillin, and 100 mg mL^−1^ streptomycin. The cells were incubated in a humified cell culture incubator with 5% CO_2_ at 37 °C. For phagocytosis and survival assays, 24 h prior to infection, 2 × 10^5^ cells were seeded in antibiotic-free medium containing 10% FCS in 24-well plates and the cells were differentiated into macrophage-like cells with 10 ng mL^−1^ phorbol 12-myristate 13-acetate (PMA). Bacteria from an over-day culture grown to an optical density at 600 nm wavelength (OD_600_) of 0.4 to 0.6 were harvested by centrifugation. A master mix of the inoculum was prepared in antibiotic-free RPMI medium with a MOI of 25 and the cells were infected by the addition of 500 µL of this solution per well. To allow the phagocytosis of bacteria, the assay plates were centrifuged for 5 min at 350× *g* and incubated in a cell culture incubator for 30 min at 37 °C with 5% CO_2_ and 90% humidity. Cells were washed with phosphate-buffered saline (PBS) and treated with 100 µg mL^−1^ gentamicin to kill extracellular bacteria. Intracellular bacteria were either recovered after 2 h by lysis of cells with 500 µL of 0.1% Triton-X100 in PBS or the cells were further incubated with medium containing a lower gentamicin concentration (10 µg mL^−1^) to analyze at later time points. To determine the number of CFUs, serial dilutions of the inoculi and lysates were plated on Columbia agar with sheep blood (Oxoid) using an Eddy Jet Version 1.22 (IUL Instruments). To calculate the percentage of viable intracellular bacteria, the ratio of bacteria used for infection and bacteria in the lysate was multiplied by 100. The survival of the bacteria was calculated by setting the 2 h value to 100% and calculating the later time points based on this value.

### 4.3. Live Cell Imaging

A density of 1.2 × 10^5^ THP-1 cells were seeded on sterile 8-well glass bottom µ-slide in antibiotic-free RPMI medium containing 10% FCS. The cells were differentiated to macrophage-like cells by the addition of 10 ng ml^−1^ PMA 24 h prior to infection. The non-adherent and dead cells were removed by washing the cells two times with PBS and fresh RPMI medium was replaced. The staining solutions were prepared in PBS with 0.1 µg mL^−1^ Hoechst 33342 and 1 µg mL^−1^ propidium iodide. The cells were stained and incubated at 37 °C in 5% CO_2_ in a humified cell culture incubator at least 20 min prior to infection with bacteria. GFP-expressing corynebacteria were harvested at an OD_600_ 0.4–0.6 and an inoculum at a MOI of 25 was prepared in PBS to infect the macrophages. Micrographs were taken at different time points and analyzed on a BZ-X710 microscope (Keyence, Neu-Isenburg, Germany). Post-processing of pictures was performed with Photoshop CS5 (Adobe, München, Germany) and ImageJ software packages.

### 4.4. Flow Cytometry

For FACS analysis, 4 × 10^5^ THP-1 cells were seeded 24 h prior to infection in 12-well plates and differentiated by the addition of 10 ng mL^−1^ PMA. Cells were infected with corynebacteria at a MOI of 25, as described above. After 5 h post-infection, cells were harvested, washed once with PBS and the supernatant with potentially detached cells was saved to collect all the cells. Then, 500 µL of cold 50 mM ethylenediaminetetraacetic acid (EDTA) in PBS was added in each well and incubated for at least 30 min at 37 °C in 5% CO_2_ in a cell culture incubator. Subsequently, the cells were removed gently with the help of cell scrapers, transferred to FACS tubes and centrifuged at 1000× *g* for 10 min. The supernatant was carefully removed and the cell pellet was resuspended in 200 µL of freshly prepared staining solution (1 µg mL^−1^ AxA5-FITC, 1 µg mL^−1^ PI, 1 µg mL^−1^ DiIC1, and 0.1 µg mL^−1^ Hoechst 33342) in Ringer’s solution (147 mM NaCl, 4 mM KCl, and 2.3 mM CaCl_2_) with 10% BSA. The samples were incubated in the dark for 25 min and subsequently 200 µL of FACS buffer (2 mM EDTA in 10% PBS) was added until analyzed by flow cytometry. To test the influence of necroptosis, 50 µM Necrostatin-1 inhibitor was added to THP-1 cells 30 min prior to infection and the procedure was followed as above. Flow cytometry was performed with a Gallios cytofluorometer (Beckman Coulter, Fullerton, CA, USA). Electronic compensation was used to eliminate bleed through fluorescence. After gating and excluding cell debris, data were analyzed using Kaluza Software version 2.1 (Beckman Coulter, Fullerton, CA, USA). The cells were classified into viable, stressed, apoptotic, and necrotic according to their staining properties and morphology.

### 4.5. Cytotoxicity Assay

The release of cytosolic lactate dehydrogenase (LDH) was measured using the cytotoxicity detection kit (LDH) according to the supplier′s instructions (Roche). To determine the cytotoxicity upon infection with corynebacteria, 100 µL supernatant of infected THP-1 cells were mixed with 2.5 µL of the provided catalyst solution and 112.5 µL of the provided dye solution in 96-well plates. The plates were incubated in the dark for 30 min and the absorbance was measured in a multimode plate reader (TECAN Infinite 200 PRO) at 490 nm with a wavelength correction set at 620 nm. The maximum LDH release was determined by treatment of the cells with 2% Triton X-100.

### 4.6. Statistical Analysis

Statistical analyses were performed using GraphPad Prism 7.0 (GraphPad, San Diego, CA, USA) software. Results were compared with two-tailed t-tests with a minimal level of significance set at *p* < 0.05. Results of all groups were compared with ANOVA column statistics and Bonferroni corrections were applied for multiple comparisons.

## Figures and Tables

**Table 1:** Strains, cell lines, and plasmids used in this study.

**Bacterial Strains**	**Description/Genotype**	**Reference/Source**
*C. glutamicum* ATCC 13032	type-strain, non-pathogenic	[37]
*C. diphtheriae* HC04	Isolated from a 7 year old female with a fatal endocarditic infection, wild-type, non-toxigenic (tox^−^)	[13,26]
*C. ulcerans* 809	Isolated from an 80 year old woman with a fatal pulmonary infection, non-toxigenic (tox^−^)	[38]
**Cell lines**	**Description**	**Reference/Source**
THP-1	Human leukemic monocytic cells	[39]
THP1-Blue NF-κB	THP-1 cells with stable integrated NF-κB inducible SEAP reporter construct	Invivogen
**Plasmids**	**Description**	**R** **eference/Source**
pEPR1-p45*gfp*	*P45*, *gfpuv*, Km^R^*, rep*, *per,* T1, T2	[40]

## Abbreviations

CFU	Colony-forming unit(s)
EDTA	Ethylenediaminetetraacetic acid
FACS	Fluorescence-activated cell sorting
FCS	Fetal calf serum
FITC	Fluorescein isothiocyanate
GFP	Green-fluorescent protein
HI	Heart infusion
LDH	Lactate dehydrogenase
MOI	Multiplicity of infection
NEC-1	Necrostatin-1
OD_600_	Optical density at 600 nm wavelength
PBS	Phosphate-buffered saline
PI	Propidium iodide
PLD	Phospholipase D
PMA	phorbol 12-myristate 13-acetate
RPMI	Roswell Park Memorial Institute
